# Evidence of assortative mating for theory of mind via facial expressions but not language

**DOI:** 10.1177/02654075221106451

**Published:** 2022-06-02

**Authors:** Emily Jackson, John Galvin, Varun Warrier, Simon Baron-Cohen, Shanhong Luo, Robin IM Dunbar, Hannah Proctor, Eva Lee, Gareth Richards

**Affiliations:** 1 School of Psychology, Faculty of Medical Sciences, 5994 Newcastle University, Newcastle upon Tyne, Tyne and Wear, UK; 2 Department of Psychology, 1725 Birmingham City University, Birmingham, West Midlands, UK; 3 Autism Research Centre, Department of Psychiatry, 2152 University of Cambridge, Cambridge, Cambridgeshire, UK; 4 Department of Psychology, 14621 University of North Carolina Wilmington, Wilmington, NC, USA; 5 Department of Experimental Psychology, 6396 University of Oxford, Oxford, UK

**Keywords:** Assortative mating, Behavioural genetics, Empathizing, Evolutionary psychology, Reading the Mind in the Eyes Test, Sex differences, Stiller-Dunbar Stories Task, Theory of mind

## Abstract

Assortative mating is a phenomenon in which romantic partners typically resemble each other at a level greater than chance. There is converging evidence that social behaviours are subject to assortative mating, though less is known regarding social cognition. Social functioning requires the ability to identify and understand the mental states of others, i.e., theory of mind. The present study recruited a sample of 102 heterosexual couples via an online survey to test if theory of mind as measured using facial expressions (Reading the Mind in the Eyes Test) or language (Stiller-Dunbar Stories Task) is associated with assortative mating. Results provide evidence of assortative mating for theory of mind via facial expressions, though there was no such effect for theory of mind via language. Assortative mating for theory of mind via facial expressions was not moderated by length of relationship nor by partner similarity in age, educational attainment, or religiosity, all variables relevant to social stratification. This suggests assortative mating for theory of mind via facial expressions is better explained by partners being alike at the start of their relationship (initial assortment) rather than becoming similar through sustained social interaction (convergence), and by people seeking out partners that are like themselves (active assortment) rather than simply pairing with those from similar demographic backgrounds (social homogamy).

## Introduction

Despite enduring popularity for the notion that “opposites attract”, evidence from empirical research more readily supports the view that “birds of a feather flock together” (Luo, 2017; McPherson et al., 2001; Montoya et al., 2008). In the context of romantic relationships, this reflects the presence of assortative mating, that is, the non-random pattern in which partners show greater similarity to one another than expected by chance. The highest within-couple correlations are typically observed for demographic variables, such as age and ethnicity, and are followed by educational attainment and attitudes; the weakest correlations are usually reported for personality traits and physical characteristics (see Luo, 2017).

### Mechanisms of assortative mating

Assortative mating can arise through various means (Luo & Klohnen, 2005). For instance, individuals may be more likely to meet and thus form a relationship if they share a similar social background (social homogamy). However, despite the strength of within-couple correlations observed for demographic variables (Blossfeld & Timm, 2003), this resemblance rarely appears to account for similarity across other characteristics. For example, assortative mating has been reported to occur within a range of populations that are relatively demographically homogeneous (Burgess & Wallin, 1943; Godoy et al., 2008; Kardum et al., 2017; Luo, 2009; Luo & Klohnen, 2005; Richards et al., 2022; Tognetti et al., 2014; Wu et al., 2020). Assortative mating could arise through convergence, i.e., couples may grow alike over time due to their interactive experiences and shared environment. Empirical findings cast doubt on this suggestion, as relationship length does not correlate with couple similarity for most variables that have been examined (Kardum et al., 2017; Luo & Klohnen, 2005; Richards et al., 2022). Furthermore, assortative mating is detectable early in relationships (Luo, 2009), indicating the presence of initial assortment. It is thus largely accepted that couple similarity is driven mainly by initial and active assortment, rather than by social homogamy and/or convergence (Luo & Klohnen, 2005).

### Social abilities and theory of mind

Social abilities may play an important role in assortative mating, as they could not only affect initial pairing but also influence interactions throughout a relationship. Indeed, there is evidence for assortative mating in social skills and characteristics related to interpersonal functioning. For instance, some studies report assortative mating effects for traits associated with autism, a spectrum of neurodevelopmental conditions characterised in part by difficulties in social interaction (Connolly et al., 2019; Constantino & Todd, 2005; Richards et al., 2022; Virkud et al., 2009). Moreover, Burleson and Denton (1992) found evidence of assortative mating for cognitive complexity, a socio-cognitive construct that enables social information processing and facilitates perspective taking (Hale & Delia, 1976). Interestingly, couples consisting of two low-scoring partners were no less satisfied than couples consisting of two high-scoring partners. The authors therefore suggested that similarity in social abilities can promote enjoyable and harmonious interactions that ultimately enhance relationship satisfaction. Such assortment is also evident in platonic relationships, with friends showing increased similarity for relevant skills such as communication ability (Burleson et al., 1992) and empathic distress (Burleson et al., 1992; Kurdek & Krile, 1982; R. L. Smith & Rose, 2011), as well as for measures of autistic traits (Faso et al., 2016; Wainer et al., 2013). These findings suggest that individuals seek relationships with others who are cognitively similar. This could be because comparable outlooks that validate one’s own beliefs are positively reinforcing (Burleson et al., 1992; Montoya et al., 2008). Alternatively, such effects could be a passive product of mating market operation (Luo, 2017). Essentially, as the most desirable partners may also be those with high levels of social skills, competition for such partners will be most intense. This could result in those with high levels of social skills being more likely than chance to pair with each other, leaving those with lower levels of social skills to choose partners from the remaining pool of potential mates characterised by lower social skills.

Couple assortment for social abilities may rely to some degree on a capacity to interpret the mind states of others and to understand that these can differ from one’s own mind state: that is, it relies on a ‘theory of mind’ (Baron-Cohen, 1995; Premack & Woodruff, 1978). Two dissociable yet related components of theory of mind have been identified: socio-cognitive and socio-perceptual (Dvash & Shamay-Tsoory, 2014; Shamay-Tsoory & Aharon-Peretz, 2007; Tager-Flusberg & Sullivan, 2000). The socio-cognitive component encompasses the ability to reason about the mental states of others (Nettle & Liddle, 2008). It is commonly assessed by measures such as the Stiller-Dunbar Stories Task (Powell et al., 2010; Stiller & Dunbar, 2007), which require participants to read socially complex short stories and then answer questions about the perspectives of characters presented within them. The socio-perceptual component of theory of mind, on the other hand, relates to the ability to rapidly detect the mental states of others based on peripheral cues such as facial expressions and body movements (Nettle & Liddle, 2008). It is commonly measured using the Reading the Mind in the Eyes Test (Baron-Cohen et al., 2001), which requires participants to identify mental states from images of the eye region of people’s faces. Socio-perceptual theory of mind therefore appears to represent the more reflexive component and is closely related to the affective system (Tager-Flusberg & Sullivan, 2000). Notably, there is also evidence for distinct ‘nice’ and ‘nasty’ theory of mind (Ronald et al., 2005), and one’s ability in each domain may be differentially associated with prosocial and antisocial outcomes (Lonigro et al., 2014). The terms ‘perceptual’ and ‘cognitive’ are not ideal in the current context since these are not easy to disentangle because the former entails the latter. Therefore, in what follows, we use different terminology that we hope is more descriptive and unambiguous: we refer to the Reading the Mind in the Eyes Test as a test of theory of mind via facial expressions and the Stiller-Dunbar Stories Task as a test of theory of mind via language.

Although some recent studies (Esménio et al., 2019; Ramezani et al., 2020) have considered theory of mind within the context of romantic relationships, we are aware of only one that has specifically examined this construct in relation to assortative mating. More specifically, Richards et al. (2021 [Study 1], 2022) reported preliminary evidence of assortative mating for theory of mind via facial expressions, with scores on the Reading the Mind in the Eyes Test being positively correlated within heterosexual couples from the UK. However, the sample size was relatively small (*n*=55 couples), the finding has not yet been independently replicated, and the study did not examine theory of mind via language. Nevertheless, as there is evidence for assortative mating in relation to constructs closely related to theory of mind via language, such as cognitive complexity (Burleson & Denton, 1992) and emotional intelligence (Śmieja & Stolarski, 2018), and because partner similarity in such characteristics appears advantageous for the formation and maintenance of interpersonal relationships, positive assortative mating can be predicted to occur.

### Hypotheses and predictions

The current study aimed to investigate within-couple similarity for theory of mind by using an online survey and was pre-registered on the Open Science Framework (https://osf.io/ja7x2). We predicted that (1) total scores for the Reading the Mind in the Eyes Test and the Stiller-Dunbar Stories Task would each be positively correlated between male and female partners in heterosexual relationships, and (2) that smaller difference scores (indicating increased similarity) for these variables would be observed for actual couples than for random male-female pairings. Additionally, we predicted that any couple similarity effects observed here would reflect initial and active assortment rather than convergence and/or social homogamy.

## Method

### Participants

We conducted an a priori power analysis using G*Power 3.1 (Faul et al., 2007, 2009) to determine the required sample size. We used an effect size estimate (*r* = 0.438) of within-couple correlation for the Reading the Mind in the Eyes Test observed by Richards et al. (2021 [Study 1]). Please note that *r* = 0.438 (Pearson’s test) was the effect size included in the preliminary report of Richards et al. (2021, Study 1), whereas, in response to a reviewer’s suggestion, Richards et al. (2022) subsequently reported *r*_s_ = 0.329 (Spearman’s test) in a more detailed analysis of the same dataset. With a requirement of 80% power, it was determined that a sample size of *n*=38 couples would be needed to observe a statistically significant effect (*p* < 0.05) using a two-tailed Pearson’s correlation. However, we aimed to collect data from *n*=100 couples (via an online survey) because (1) we intended to negate the loss of statistical power associated with missing data (i.e., some participants might not complete all required measures), (2) we aimed to protect against “winner’s curse” and effect size inflation associated with relatively small sample sizes, and (3) because the dataset was additionally used to examine assortative mating in relation to Dark Triad personality traits, variables which have been reported to show smaller within-couple correlations (results from this additional study were also pre-registered and will be presented separately). Ethical approval was obtained from the Faculty of Medical Sciences Research Ethics Committee, Newcastle University (reference number: 7573/2020).

The study was based in Newcastle upon Tyne, UK, and participants comprised an opportunity sample of heterosexual couples recruited through word of mouth and snowball sampling. To take part, participants were required to be aged 18 years or over and to be in a heterosexual relationship at the time. Data were available for *n*=104 complete couples (i.e., those couples for which both partners completed at least one of the theory of mind measures), although two couples that each included one non-binary member were not included in further analysis. As participants were asked to specify their sex (but not gender identity), these couples were removed because it is difficult to determine whether this item was interpreted correctly. For those *n*=102 couples that were retained, the mean age of males was 36.64 years (*SD* = 17.83 [median = 25.5, range = 18–84]), and that for females was *M* = 35.55 years (*SD* = 17.62 [median = 24.0, range = 18–80]). The mean length of relationship was 12.91 years (*SD* = 14.99), and most participants were UK residents (males: *n* = 95, 93.14%; females: *n* = 97, 95.10%). The majority (males: *n* = 65, 63.73%; females: *n* = 58, 56.86%) were employed and a little over one third were students (males: *n* = 33, 32.35%; females: *n* = 44, 43.14%).

Most participants were White/Caucasian (males: *n* = 89, 87.25%; females: *n* = 87, 85.29%), and relatively few reported being of other ethnicities: Asian/Asian British (males: *n* = 10, 9.80%; females: *n* = 10, 9.80%), Black/Black British (males: *n* = 0, 0.00%; females: *n* = 0, 0.00%), Black/other (males: *n* = 1, 0.98%; females, *n* = 0, 0.00%), Chinese (males: *n* = 2, 1.96%; females: *n* = 1, 0.98%), Hispanic/Latino (males: *n* = 0, 0.00%; females: *n* = 3, 2.94%), Middle/Near Eastern (males: *n* = 0, 0.00%; females, *n* = 0, 0.00%), mixed ethnicity (males: *n* = 0, 0.00%; females, *n* = 2, 1.96%) (note that participants could select more than one ethnicity category). Slightly more than half (55.88%) reported living with their partner; approximately one third were married (males: *n* = 38, 37.26%; females: *n* = 39, 38.24%), few were engaged (males: *n* = 2, 1.96%; females, *n* = 2, 1.96%), and most were not married (males: *n* = 62, 60.78%; females: *n* = 61, 59.80%). It is unclear why 39 females, but only 38 males, reported being married. Although speculative, this could reflect one of the females remaining legally married to a previous partner from whom she had separated. The sample as a whole reported relatively high academic attainment, as follows: no qualifications (males: *n* = 2, 1.96%; females: *n* = 0, 0%); completed GCSE level (or equivalent) (males: *n* = 9, 8.82%; females, *n* = 8, 7.84%); completed A level or Access Course (or equivalent) (males: *n* = 40, 39.22%; females: *n* = 42, 41.18%); Bachelor’s Degree (males: *n* = 38, 37.26%; females: *n* = 33, 32.35%); Master’s Degree (males: *n* = 9, 8.82%; females: *n* = 16, 15.69%); Doctorate Degree (males: *n* = 4, 3.92%; females: *n* = 3, 2.94%).

### Design

The present study employed a correlational design to examine within-couple correlations/similarity for scores on the Reading the Mind in the Eyes Test (theory of mind via facial expressions) and the Stiller-Dunbar Stories Task (theory of mind via language).

### Materials

#### Demographic questionnaire

Participants were administered demographic questions to determine their age, sex, country of residence, ethnicity, educational attainment, living status (i.e., cohabiting with partner or not), relationship length, and marital status.

#### Stiller-Dunbar Stories Task (theory of mind via language)

We used a revised version of Stiller and Dunbar’s (2007) Stories Task, similar to that of Powell et al. (2010), to measure theory of mind via language. This comprises five short stories, each approximately 200 words long, which depict various social scenarios involving interactions between three to five characters. After reading each story, participants were asked to answer true or false questions relating to its content. These assessed the ability to consider various levels of perspectives ranging from two to six levels of intentionality, where two requires the participant to track the perspective of one character and six requires the participant to track five embedded levels of perspective. Factual questions used in Stiller and Dunbar’s (2007) original study were omitted here to reduce the length of the questionnaire and because we did not set out to investigate memory ability. Participants were advised to answer as quickly as possible and to refrain from changing their answers once selected. A total score was calculated from the number of correct responses and could range from 0 to 49. Cronbach’s alpha indicated satisfactory internal consistency (α = 0.601).

#### Reading the Mind in the Eyes Test (theory of mind via facial expressions)

To test theory of mind via facial expressions, we used the Reading the Mind in the Eyes Test (Baron-Cohen et al., 2001). This assesses the ability to correctly identify mental states from pictures of the eye region of people’s faces. More specifically, participants were shown 36 images of eyes (18 male and 18 female) portraying various expressions. Each image was presented individually along with four adjectives (one target word and three foil words) describing complex mental states (e.g., ‘contemplative’, ‘encouraging’, ‘despondent’, ‘flustered’). Participants were instructed to select the adjective they believed best described the mental state depicted in each picture (a unique set of adjectives was included for every image). They also had access to dictionary definitions of the adjectives and were advised to respond based on instinct and to refrain from changing any answers once they had been selected. A total score was calculated as the number of correct responses across 36 trials. Internal consistency for the Reading the Mind in the Eyes Test in the present study was satisfactory (α = 0.713).

#### General Religiosity Measure

As large social homogamy effects may occur in relation to religious beliefs and practices, we employed the General Religiosity Measure (Saroglou & Galand, 2004; Saroglou & Muñoz-García, 2008). This provides a 3-item indication of intrinsic (‘general’) religiosity, i.e., the strength of a person’s religious beliefs or feelings and how important those beliefs are in their life, regardless of which religion they may (or may not) follow. Each item is answered on a 7-point Likert scale, with higher scores indicating higher religiosity. Cronbach’s alpha for the present study indicated satisfactory internal consistency (α = 0.752).

#### Procedure

Adults aged 18 years or over and known to be in a heterosexual relationship were contacted by the researchers and asked to participate in an online survey (hosted by Qualtrics) investigating cognitive and perceptual abilities of romantic partners. They were given information about the study, asked to provide consent before proceeding further, and were debriefed on completion. Participants were advised to complete the survey independently, in one sitting, and to avoid discussing any of the tasks with their partner during the study. They were also offered an opportunity to enter a prize draw for a £25 Amazon voucher. The first member of each couple to complete the study was assigned a unique couple identification number, which they were asked to share with their partner. This was used to pair each participant’s data with those of their partner and was subsequently deleted to maintain anonymity.

#### Data analysis

Paired samples *t* tests were conducted to test for within-couple sex differences in the theory of mind measures. We then used Pearson’s tests to examine within couple correlations for variables relevant to social homogamy (age, intrinsic religiosity, and educational attainment [note that, due to the ordinal coding of the educational attainment variable, a Spearman’s test was used in this case]).

We conducted both variable-centred and couple-centred analyses to examine within-couple correlation/similarity for theory of mind variables (Luo & Klohnen, 2005). For the variable-centred analysis, we used Pearson’s tests to determine the strength and direction of correlation within couples for the Stiller-Dunbar Stories Task total score and the Reading the Mind in the Eyes Test total score. To conduct the couple-centred analysis, we first computed unsigned (i.e., absolute) difference scores (for both the Reading the Mind in the Eyes Test and the Stiller-Dunbar Stories Task, respectively) for each couple by subtracting the standardised score of the female from the standardised score of the male, and removing the sign. We next computed difference scores for random pairings by matching every male with every female in the dataset that was not his partner (and therefore also matching every female with every male in the dataset that was not her partner), taking the average, and removing the sign. We used paired-samples *t* tests to compare the unsigned difference scores for actual couples with those computed for random pairings. Smaller difference scores for actual couples than random couples indicate a greater level of partner resemblance than expected by chance. These two types of analysis differ in a subtle way. Whereas the variable-centred approach is used to determine whether a high or low score in one person is a predictor of whether their partner has a high or low score on the same measure, the couple-centred approach is used to determine whether actual couples’ scores are more similar than chance assuming a pattern of random mating.

If a statistically significant (i.e., *p* < 0.05, two-tailed) within couple-correlation (i.e., from the variable-centred approach) was detected for a theory of mind measure, we correlated the within-couple (unsigned) difference score for that measure with the length of relationship. The idea here is that if smaller difference scores (i.e., greater similarity) are associated with longer relationships (indicated by a significant negative correlation) then partner resemblance may be better explained by convergence than by initial assortment. We also correlated the unsigned within-couple difference score for the theory of mind measure with the within-couple difference scores for three variables relevant to social homogamy (Luo & Klohnen, 2005): age, educational attainment, and intrinsic religiosity. In this case, a significant positive correlation would indicate that couples which are similar in their demographic background are more similar for theory of mind than expected by chance, and so would provide evidence of social homogamy rather than active assortment.

Data analysis was conducted in RStudio (Version 1.3.1073), and all tests were two-tailed. A criterion of *p* < 0.05 was used to indicate statistical significance, and effect sizes were interpreted in accordance with generally accepted criteria (Cohen, 1988). The dataset and R code used to run the analysis are available on the Open Science Framework (https://osf.io/bk5c3/).

## Results

### Sex differences

Consistent with observations from previous studies (Baron-Cohen et al., 2015; Stiller & Dunbar, 2007), paired samples *t* tests revealed that females scored significantly higher than their male partners on both the Reading the Mind in the Eyes Test and the Stiller-Dunbar Stories Task (Table 1).

**Table 1.** Descriptive statistics and sex differences for theory of mind variables.

	Males		Females		Difference			
	M	SD	M	SD	t	df	p	d
Reading the Mind in the Eyes Test	25.48	4.87	26.87	4.52	−2.468	95	0.015	−0.301
Stiller-Dunbar Stories Task	35.19	4.23	37.54	3.75	−4.284	99	<0.001	−0.591

*Note*. *M* = mean. *SD* = standard deviation.

### Variable-centred analysis

**Figure 1.**
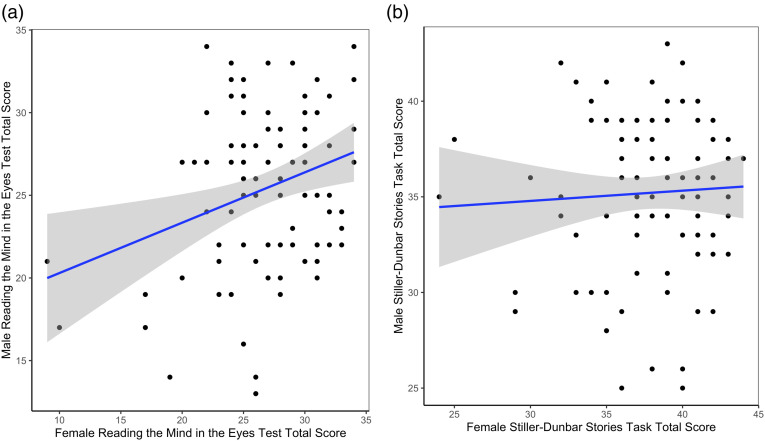
Scatterplots showing within-couple correlations for (**a**) Reading the Mind in the Eyes Test (theory of mind via facial expressions) and (**b**) Stiller-Dunbar Stories Task (theory of mind via language).

A strong positive correlation was observed between partners’ ages, *r* (100) = 0.980, *p* < .001, and small-to-moderate within-couple correlations were present for educational attainment *r*_*s*_ = 0.426, *p* < 0.001, and intrinsic religiosity, *r* (100) = 0.445, *p* < 0.001. As predicted, partners’ scores on the Reading the Mind in the Eyes Test were significantly positively correlated, *r* (94) = 0.284, *p* = 0.005 (Figure 1a); however, there was no such effect for the Stiller-Dunbar Stories Task, *r* (98) = 0.048, *p* = 0.635 (Figure 1b). Although not specified in our pre-registered analysis plan, we used Pearson’s correlations to examine the relationship between theory of mind via facial expressions and theory of mind via language. Scores on the Reading the Mind in the Eyes Test and the Stiller-Dunbar Stories Task were significantly and moderately positively correlated in both males, *r* (95) = 0.262, *p* = 0.010, and females, *r* (97) = 0.461, *p* < 0.001. A Fisher’s *r*-to-*z* test showed that the difference in strength for these correlations was not statistically significant, *z* = −1.59, *p* = 0.112 (two-tailed).

### Couple-centred analysis

**Figure 2.**
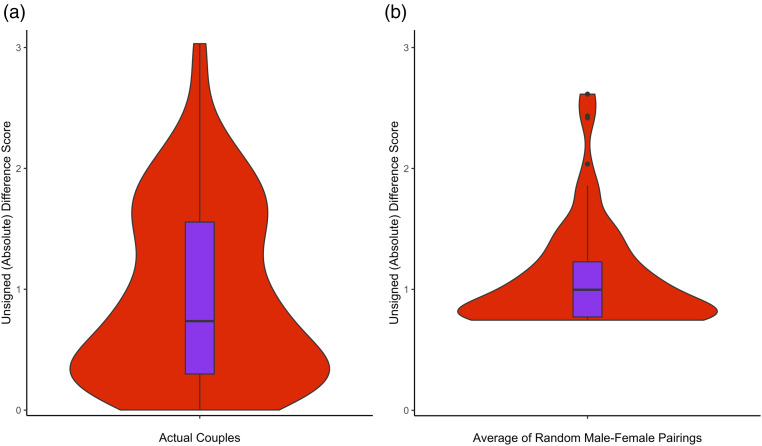
Violin boxplots showing the distribution of unsigned difference scores for the Reading the Mind in the Eyes Test (theory of mind via facial expressions) for (**a**) actual couples and (**b**) the average of all other male-female pairings possible within the dataset.

The unsigned difference score for the Reading the Mind in the Eyes Test was smaller for actual couples (*M* = 0.944, *SD* = 0.73) than the average calculated from all other male-female pairings possible within the dataset (*M* = 1.096, *SD* = 0.426), *t* (95) = −2.218, *p* = 0.029, *d* = −0.243 (see Figure 2a for violin boxplot). In accord with findings from the variable-centred analysis, within-couple difference scores for the Stiller-Dunbar Stories Task were not significantly different (actual couples: *M* = 1.088, *SD* = 0.830; average of other male-female pairings: *M* = 1.096, *SD* = 0.407), *t* (99) = −0.107, *p* = 0.915, *d* = −0.011 (Figure 2b).

### Initial assortment versus convergence, and active assortment versus social homogamy

As a significant within-couple correlation was observed for the Reading the Mind in the Eyes Test, we correlated the unsigned within-couple difference scores for this variable with length of relationship to test for evidence of a convergence effect. No significant correlation emerged, *r* (94) = −0.085, *p* = 0.408, suggesting that couple similarity for this variable is better explained by initial assortment. To test for social homogamy, we then correlated the unsigned within-couple difference scores for the Reading the Mind in the Eyes Test with the unsigned within-couple difference scores for age, educational attainment, and intrinsic religiosity. No significant effects were observed (age: *r* [94] = −0.039, *p* = 0.707; educational attainment, *r* [94] = 0.127, *p* = 0.219; intrinsic religiosity, *r* [94] = −0.052, *p* = 0.616), suggesting that couple resemblance for this measure is better explained by active assortment.

As it can be challenging to disentangle effects relating to phenotypic and social homogamy (Heath & Eaves, 1985), and because within-couple correlations for educational attainment may occur simply because people with similar levels of educational attainment are more likely than chance to meet due to assorting into similar workplaces etc., we conducted a further (not pre-registered) analysis to ascertain whether controlling for educational attainment would attenuate the within-couple correlation for Reading the Mind in the Eyes Test scores. More specifically, we performed a multiple linear regression analysis with male Reading the Mind in the Eyes Test score as outcome, female Reading the Mind in the Eyes Test score as predictor, and male educational attainment and female educational attainment as covariates. Female Reading the Mind in the Eyes Test score remained a significant predictor of male Reading the Mind in the Eyes Test score (*b* = 0.303, *p* = 0.006), and there were no effects of male educational attainment (*b* = 0.411, *p* = 0.448) or female educational attainment (*b* = −0.783, *p* = 0.151).

## Discussion

The present study aimed to examine couple similarity for theory of mind via facial expressions and language. In line with our hypothesis, theory of mind ability as measured by the Reading the Mind in the Eyes Test showed a positive correlation between partners. We also observed that actual couples were more alike for this variable (i.e., they had smaller difference scores) compared to the average of all other male-female pairings possible within the dataset. Further analysis revealed that the within-couple correlation for theory of mind via facial expressions was not moderated by relationship length nor by partner similarity in age, educational attainment, or intrinsic religiosity, which suggests that the association is better explained by initial and active assortment than by convergence or social homogamy. Although these effects were in accord with our predictions, we did not observe any evidence of assortative mating for theory of mind via language, as measured by the Stiller-Dunbar Stories Task.

The positive correlation observed between partners’ theory of mind ability via facial expressions directly replicates the earlier finding of Richards et al. (2021 [Study 1], 2022); more specifically, both studies report evidence from variable-centred and couple-centred analyses for increased couple similarity in performance on the Reading the Mind in the Eyes Test. Furthermore, these studies found that the within-couple correlation for this measure was better explained by initial and active assortment than by convergence or social homogamy. As Richards et al. (2021 [Study 1], 2022) administered the Reading the Mind in the Eyes Test in a lab study whereas here we utilised online survey methodology, the effect appears to be replicable across settings. These findings also align with wider research in which positive assortment has been reported for characteristics related to socio-perceptual theory of mind ability (Kardum et al., 2017; Śmieja & Stolarski, 2018). It should however be noted that as greater than chance similarity between individuals is found throughout social strata, i.e., not only within the context of romantic relationships (McPherson et al., 2001), our use of snowball sampling will likely have resulted in participants being more alike than chance. Although pairings between males and females who were not in relationships together (i.e., for the couple-centred analysis) may therefore not be considered truly random, this would act to reduce the size of effect observed, making it more difficult to produce evidence against the null hypothesis.

When considering why positive assortment occurs for theory of mind via facial expressions, it is possible that similarity in social abilities leads to more satisfying relationships. For example, those who perceive themselves as having similar levels of empathy (Kimmes et al., 2014) and emotional intelligence (L. Smith et al., 2008) as their partners report higher relationship satisfaction than those who perceive themselves as being disparate in these domains. Additionally, theory of mind ability via facial expressions is positively correlated with prosocial orientation (Declerck & Bogaert, 2008). This is notable because prosocial behaviours exhibit spousal resemblance and are likely beneficial in terms of relationship and reproductive outcomes (Tognetti et al., 2014).

Turning to theory of mind via language, the lack of partner correlation observed in the current study is at odds with previous research reporting evidence of assortment for closely related abilities (Burleson & Denton, 1992; Constantino & Todd, 2005; Richards et al., 2022; Śmieja & Stolarski, 2018). Furthermore, as theory of mind via facial expressions and language were moderately positively correlated, and theory of mind via facial expressions showed evidence of positive assortment, we might expect theory of mind via language to show a similar pattern simply due to it being a facet of the same underlying construct (i.e., theory of mind as a whole). While these contrasting findings support the notion that these two aspects of theory of mind are related yet dissociable abilities (Dvash & Shamay-Tsoory, 2014; Shamay-Tsoory & Aharon-Peretz, 2007; Tager-Flusberg & Sullivan, 2000), they contradict our hypothesis because we expected both to show evidence of assortative mating.

Statistically significant effects for theory of mind via facial expression (but not via language) may reflect the evolutionarily ancient selection pressure by which humans (as well as relevant ancestral species) have interacted face-to-face in mate choice contexts (Rhodes, 2006). This could also have enabled humans’ capacity to infer social information from faces, and indeed for human faces to evolve in ways that facilitate communication of such information (Zebrowitz & Montepare, 2006). Assortative mating may arise because individual preferences for mates are often contingent on identifying those with a consonant mating strategy. Such processes can be conceptualised within an affordance-management framework, i.e., the idea that the purpose of the mind “is to identify and then manage the opportunities and threats in the physical and social environment” (Neuberg et al., 2020, p. 247) and generate behaviour that maximises opportunities and minimises threats. Consistent with this is the finding that individuals can accurately infer mating intentions from facial cues, and that these inferences are more apparent in the faces of men than women (Antar & Stephen, 2021). Notably, interest in monogamous reproductive strategies predicts women’s preferences for men’s faces indicating high levels of agreeableness (Brown et al., 2019 [though note effect was marginally significant, *p* = 0.090]), and low levels of extraversion (Brown & Sacco, 2017), masculinity (Kruger, 2006) and narcissism (Marcinkowska et al., 2015).

It may also be important to consider the nature of abilities. Abilities are internal attributes, meaning that for assortment to occur, the ability must be expressed externally in a consistent and reliable way. Indeed, it has been suggested that within-couple correlations for such variables may be relatively small simply due to the difficulties inherent with accurately assessing them (Śmieja & Stolarski, 2018). It is also worth considering that theory of mind via facial expressions and language rely on distinct neural substrates (Dvash & Shamay-Tsoory, 2014). This makes it noteworthy that R. L. Smith and Rose (2011) reported empathic distress (an affective response) but not social perspective-taking to be significantly positively correlated within early-adolescent friendship dyads, a pattern of findings that somewhat mirrors that of the present study. The connection between theory of mind via facial expressions and the neural substrates involved in emotional processing (Dvash & Shamay-Tsoory, 2014) could also suggest that relevant behavioural practices increase visibility of this skill more effectively than is possible for theory of mind via language. Thus, self-resemblance in this ability may be more easily detected and evaluated by potential partners, making it more likely to undergo assortative mating.

A further consideration is the types of behaviour that are associated with abilities. For instance, while both theory of mind components are positively correlated with prosocial behaviour in children (Imuta et al., 2016), differences emerge when considering their relation to antisocial tendencies. Sutton et al. (1999) found socio-cognitive theory of mind to correlate positively with bullying behaviours, suggesting that an ability to understand the mind states of others may give rise to the capacity for manipulative aggression. Socio-perceptual theory of mind, on the other hand, has been found to correlate negatively with conduct problems (Sharp, 2008). Machiavellianism, a personality trait characterised by manipulative behaviour and a lack of interpersonal affect (and subject to assortative mating; Kardum et al., 2017), is also negatively correlated with socio-perceptual theory of mind in young adults (Ali & Chamorro-Premuzic, 2010), but is not characterised by deficits in socio-cognitive theory of mind (Paal & Bereczkei, 2007). Thus, it appears that the socio-perceptual component of theory of mind, likely due to its links with affect and empathy (Eisenberg & Miller, 1987), more consistently predicts prosocial behaviour and a lack of antisocial behaviour, whereas socio-cognitive theory of mind correlates positively with both. Furthermore, different facets of theory of mind (e.g., ‘nice’ and ‘nasty’ theory of mind; Ronald et al., 2005) may relate differently to prosocial and antisocial behaviour (Lonigro et al., 2014). Such added complexity could make detecting couple assortment for overall socio-cognitive theory of mind difficult, as it is not reliably or consistently expressed.

It should be noted that the Stiller-Dunbar Stories Task is designed to assess the upper limit of an individual’s ability (Stiller & Dunbar, 2007). Thus, if an individual’s maximal theory of mind via language does not reliably reflect the ability level displayed throughout daily life, it is unlikely that assortment for maximal ability will occur (unless as a by-product of assortment for some other, associated trait). Importantly, Keysar et al. (2003) found that adults do not routinely use the full extent of their socio-cognitive theory of mind ability, and so we may not have correlated the level at which partners’ socio-cognitive theory of mind is used throughout daily life. This may explain why the present findings differ from those of previous studies. For example, Burleson and Denton (1992) found evidence of assortative mating for cognitive complexity, an ability closely related to socio-cognitive theory of mind (Hale & Delia, 1976), when this was assessed based on participants’ written descriptions of peers. Thus, rather than maximal ability, this task likely assesses individuals’ natural level of cognitive complexity, and so the level that is expected to be most visible to others. Similarly, couple-similarity has been observed for abilities closely related to socio-cognitive theory of mind when assessed via spouse-report (Constantino & Todd, 2005; L. Smith et al., 2008). While spouse estimations may not accurately reflect underlying ability, they are likely based on the ability level that is evident through partners’ behaviour. Thus, even if partners are not matched in their maximal socio-cognitive theory of mind ability, they may be similar in the way and extent to which this ability is typically deployed. Future research should therefore investigate couple assortment for the level at which socio-cognitive theory of mind is most routinely used.

Although there are notable strengths to the current research, such as the pre-registered hypothesis and analysis plan, as well as the sample size having been determined via a priori power calculation, there are also some limitations that should be acknowledged. First, we omitted the memory-based (i.e., non-theory of mind) items from the Stiller-Dunbar Stories Task, meaning that these could not be used to assess whether attentional effort was provided to the reading of the vignettes. Additionally, although participants were asked not to confer with their partner when completing the study, the online nature of the research made it impossible to verify whether this request was followed. However, as conferring might be expected to increase rather than decrease similarity in responding, this appears unlikely to have been a problem. That is because the within-couple correlation for the Reading the Mind in the Eyes Test observed here (*r* = 0.284) was slightly smaller than that of the lab-based study of Richards et al. (2021 [Study 1], 2022) (*r*_s_ = 0.329 [*r* = 0.438]) which used the same measure. This observation could also be relevant in explaining why assortative mating effects were observed for the Reading the Mind in the Eyes Test but not for the Stiller-Dunbar Stories Task. More specifically, the former may be a more engaging task, with the latter requiring more cognitive effort, and it may be particularly difficult to hold participants’ attention for tasks requiring considerable amounts of reading in online studies. Further research should therefore examine whether assortative mating is present for socio-cognitive theory of mind tasks when administered in lab settings. The validity of the Reading the Mind in the Eyes Test itself has also been questioned, with some researchers suggesting it to operate as a measure of emotion perception rather than theory of mind (Kittel et al., 2022). Considered alongside the observation that no couple similarity effects were observed for the Stiller-Dunbar Stories Task, an alternative interpretation of the current study’s main findings is that assortative mating does not occur for theory of mind itself, but instead acts upon the closely related concept of emotion recognition. Further studies will be required to test between these hypotheses.

The current study is limited in that it did not explicitly consider gender identity, sexual orientation or disability status, variables which could be examined by future research. The sample examined also predominantly represents Western, Educated, Industrialised, Rich, and Democratic (WEIRD) individuals/societies, and so may not be generalisable to the human condition writ large (Henrich et al., 2010). This is particularly relevant in the context of assortative mating, as for most of evolutionary history, *homo sapiens* has lived in relatively small and homogeneous groups in which marital pairings would often be initiated to forge/maintain political alliances rather than due to the active preferences of those involved (Chagnon, 1983). In the developed world, however, such processes are less common, and most people live in large and heterogeneous settlements, and so have access to a vastly increased pool of potential mates. The rise of Internet dating has also facilitated active assortment (Lee, 2016), meaning that findings from studies of present-day WEIRD populations, albeit informative regarding mate selection in certain populations of modern humans, may not be an accurate reflection of our evolutionary past. Future research should therefore aim to examine similarity/dissimilarity of assortative mating processes within pre-industrial societies (Godoy et al., 2008).

## Conclusions

The current study provides evidence consistent with there being positive assortative mating for theory of mind via facial expressions but not for theory of mind via language. We suggest that inherent differences in the way these abilities are externally expressed may explain the contrasting findings. It could be that the level of this skill most routinely displayed during daily life is not estimated accurately by measures designed to determine a person’s maximal ability, or simply that the Reading the Mind in the Eyes Test is a more engaging task than the Stiller-Dunbar measure when administered in an online setting. Further research is therefore required to explore these possibilities, and to determine whether assortative mating occurs for different aspects of theory of mind within non-WEIRD populations.

## Open research statement

As part of IARR’s encouragement of open research practices, the author(s) have provided the following information: This research was pre-registered. The aspects of the research that were pre-registered were the hypothesis/predictions and the statistical analysis plan. The registration was submitted to the Open Science Framework (https://osf.io/ja7x2). The data used in the research are publicly posted on the Open Science Framework (https://osf.io/bk5c3/). The materials used in the research are publicly available other than the modified version of the Stiller-Dunbar Stories Task, which is available upon request by emailing gareth.richards@ncl.ac.uk.
